# What is the femoral shortening osteotomy in THA for congenital high hip dislocation with the lowest complication rate? A systematic review

**DOI:** 10.1530/EOR-2024-0146

**Published:** 2026-02-04

**Authors:** Carlo Casciaro, Corrado Rampulla, Silvia Bargeri, Stefania Guida, Luigi Zagra

**Affiliations:** ^1^ Fondazione Policlinico Universitario Campus Bio-Medico, Roma, Italy; ^2^ Research Unit of Orthopaedic and Trauma Surgery, Department of Medicine and Surgery, Università Campus Bio-Medico di Roma, Roma, Italy; ^3^ ASP Siracusa, PO Lentini, Unit of Orthopaedic and Trauma Surgery, Lentini, Italy; ^4^ IRCCS Istituto Ortopedico Galeazzi, Unit of Clinical Epidemiology, Milan, Italy; ^5^ Hip Department, IRCCS Istituto Ortopedico Galeazzi, Milan, Italy; ^6^ Vita Salute San Raffaele University, Milan, Italy

**Keywords:** femoral shortening osteotomy, congenital high hip dislocation, Crowe IV, total hip arthroplasty

## Abstract

**Purposes:**

- Femoral shortening osteotomy is a promising surgical intervention for patients with the sequelae of high hip dysplasia who need a THA. It offers potential benefits such as preserving limb length, reducing joint stiffness, and preventing neurovascular damage. However, it is considered a complex procedure with a high risk of complications. Various techniques are employed, and their relative safety remains unclear. What is the prevalence of complications (e.g. non-union and dislocations) and rates of revision associated with different techniques of femoral shortening osteotomy?

**Methods:**

- A systematic review was conducted according to the PRISMA guidelines. Multiple databases were searched for studies reporting complications of various femoral shortening osteotomies. Two independent reviewers selected studies, extracted data, and assessed bias. Proportional meta-analysis was employed to estimate non-union rates, while other complications and revisions were described using alluvial diagrams. The study has been registered in the PROSPERO database (CRD42023488761).

**Results:**

- In total, 53 studies (comprising 1,925 hips undergoing osteotomy) were included. The transverse subtrochanteric shortening osteotomy emerged as the most frequently utilized technique. There was an overlapping prevalence of non-union rates among the different osteotomy techniques, ranging from 0% (step-cut osteotomies) to 2% (transverse osteotomies). A higher non-union prevalence was observed with cemented stems in transverse osteotomies (4%, 95% CI: 0–9%). Dislocation rates were similar among techniques. The rate of stem aseptic loosening ranged from 7.14% (Z osteotomy) to 0% (step-cut and V-shaped osteotomies). Oblique osteotomy exhibited the highest infection rate (2.63%).

**Conclusion:**

- Overall, comparable rates of non-union were observed across osteotomy techniques. Heterogeneous results for other complications showed a low risk for all osteotomies.

**Level of evidence:**

Level IV, therapeutic study.

## Introduction

Total hip arthroplasty (THA) is commonly chosen for patients with symptomatic hip arthritis resulting from hip dysplasia (1). The anatomical irregularities linked to hip dysplasia (shallow acetabulum, with undercoverage of the femoral head and insufficiency of the anterior acetabular wall, hypoplasia of the femoral head and metaphysis, short neck, and excessive anteversion) add complexity to the THA procedure. In most severe cases (Crowe type IV), the femoral head is not centered in the ‘true’ acetabulum nor in the superior rim but is dislocated superiorly and lies in contact with the hemipelvis, where the stress forces can, in some cases, generate a new ‘false’ acetabulum (2, 3). In instances of Crowe type IV, it becomes crucial to reposition the hip into the anatomical center of rotation to ensure long-lasting results and proper functioning of the abductor muscles (4, 5). However, this repositioning increases the risk of complications due to limb lengthening. Lengthening beyond 4 cm can jeopardize the integrity of neurovascular structures (6, 7).

Furthermore, stretching of periarticular structures (muscles, tendons, and ligaments) can result in joint stiffness and premature implant loosening (8). To mitigate these tension forces and to reduce the risk of neurological traction injury and leg length discrepancy, femoral shortening osteotomy is beneficial (9). This procedure can be performed at various anatomical levels, such as subtrochanteric (the most used) (9, 10, 11, 12, 13, 14), middle shaft (less commonly used) (15), and distal (16) levels. Different surgical techniques of subtrochanteric osteotomy, including Z-shaped (17, 18, 19), transverse (12, 20, 21), oblique (22), and chevron-shaped (23, 24, 25) osteotomies, have their own advantages and disadvantages. While transverse osteotomy is a relatively straightforward technique, concerns about rotational stability may arise (10, 26) with the need to add internal fixation. The oblique subtrochanteric osteotomy enhances rotational stability and promotes bone healing by maximizing the contact surface of the fragments (22, 27). Alternate surgical techniques provide excellent stability and increased bone contact area but demand more technical expertise and complexity (28). Numerous researchers have shared their findings and viewpoints on this matter, showing their results using a single technique, yet only a handful have undertaken a direct comparison.

Furthermore, individual clinical trials frequently lack sufficient statistical power due to the low number of cases of the single series and may not be broadly applicable. In any case, shortening osteotomy and THA in high hip dislocation are considered complex procedures with a high risk of complications. Consequently, we aim to assess the prevalence of complications and relative revisions in different techniques of femoral shortening osteotomy to understand which could be the safest.

## Materials and methods

### Study design

This systematic review follows the reporting guidelines for Meta-analyses of Observational Studies in Epidemiology (MOOSE) checklist (29). The study protocol is registered in PROSPERO (CRD42023488761).

### Eligibility criteria

The inclusion criteria for this review were prospective or retrospective clinical studies with or without a control group focused on patients with a documented radiographic evaluation of developmental hip dysplasia (DDH) with Crowe type IV undergoing any femoral shortening osteotomy technique associated with THA by reporting the success or failure of intervention outcomes and complications. The primary outcomes were the rate of non-union of different types of osteotomy and the rate of implant revision for non-union of osteotomy. The secondary outcomes were the rate of dislocations, intraoperative fractures, periprosthetic fractures, infections, stem aseptic loosening, neurological and vascular complications, heterotopic ossifications, clinical outcome assessment, and revision related to each complication.

Exclusion criteria comprised case reports, surgical techniques, systematic reviews, expert opinions, studies with fewer than 10 hips, and studies with a minimum follow-up duration of less than 12 months.

### Search strategy

In June 2024, two authors (CC and CR) systematically searched PubMed, the Cochrane Database of Systematic Reviews, and the Scopus database, and the Embase database. The entire search strategy is reported in Supplementary Material, Appendix 1 (see section on Supplementary materials given at the end of the article).

### Study selection

An independent collaboration of the two authors, CC and CR, characterized the review process, with each maintaining blindness to the other’s choices regarding the inclusion of studies. Instances of discord in individual assessments were resolved through consultation with a third author, LZ. Subsequently, a manual search of the reference lists of the articles was conducted to identify additional potential inclusions.

### Data extraction

The following data were extracted: first author, study design, level of evidence (according to the tool provided by Clinical Orthopaedics and Related Research^®^), the number of patients and hips, age, gender, follow-up duration, Crowe classification, type of stem, type of osteotomy, length of the osteotomy segment, and pre- and postoperative presence of Trendelenburg. Intraoperative complications were characterized by sciatic and/or femoral nerve palsy, intraoperative periprosthetic fracture, deep venous thrombosis, or dislocation. Postoperative complications included non-union (pseudoarthrosis) of the osteotomy, implant aseptic loosening, infection, periprosthetic fracture, and dislocation. Clinical outcomes, preoperative and postoperative Harris Hip Scores, postoperative leg length discrepancy, implant revision, and heterotopic ossification were recorded. Data were collected in Microsoft Excel (Microsoft Corporation, USA). The two authors (CC and CR) independently performed data extraction, resolving discrepancies by a third author (LZ), who double-checked the final data. Missing data in the spreadsheet were labeled as not applicable.

### Quality assessment

Two authors (CC and CR) independently assessed the quality of the studies. In case of discordance, we consulted with a third author (LZ) to reach a consensus. We independently evaluated the quality of reports and methodological quality of studies by applying the methodological index for non-randomized studies scoring system (30) for non-randomized studies, where the ideal overall score is 16 for non-comparative studies and 24 for comparative studies. Regarding the adequacy of follow-up, 1 point was awarded for a follow-up of <1 year and 2 points were awarded for a follow-up of >1 year.

### Statistical analysis

We estimated the prevalence of non-union rate performing a proportional meta-analysis to indirectly compare different osteotomy types (e.g. transverse, oblique, and step-cut) using their 95% confidence intervals (CIs). We used ‘metaprop’ (31) in Stata software, calculating the pooled estimate after the Freeman–Tukey double arcsin transformation (32) to stabilize the variance. The forest plot presented the overall EStimated prevalence (ES) for non-union rate as specific proportions (the number of events on the total number of hips) in each osteotomy type subgrouped by cementless, cemented, or mixed variables. Heterogeneity between studies was assessed with Higgins *I*^2^ defined as low if *I*^2^ < 25%, moderate if *I*^2^ is between 25 and 50%, and substantial if *I*^2^ >50% (33). For the secondary outcomes, we descriptively reported results by alluvial diagrams to show the number of complications and implant revisions in each osteotomy type. RAWGraph (34) was used to perform analyses.

## Results

### Study selection

**Figure 1:**
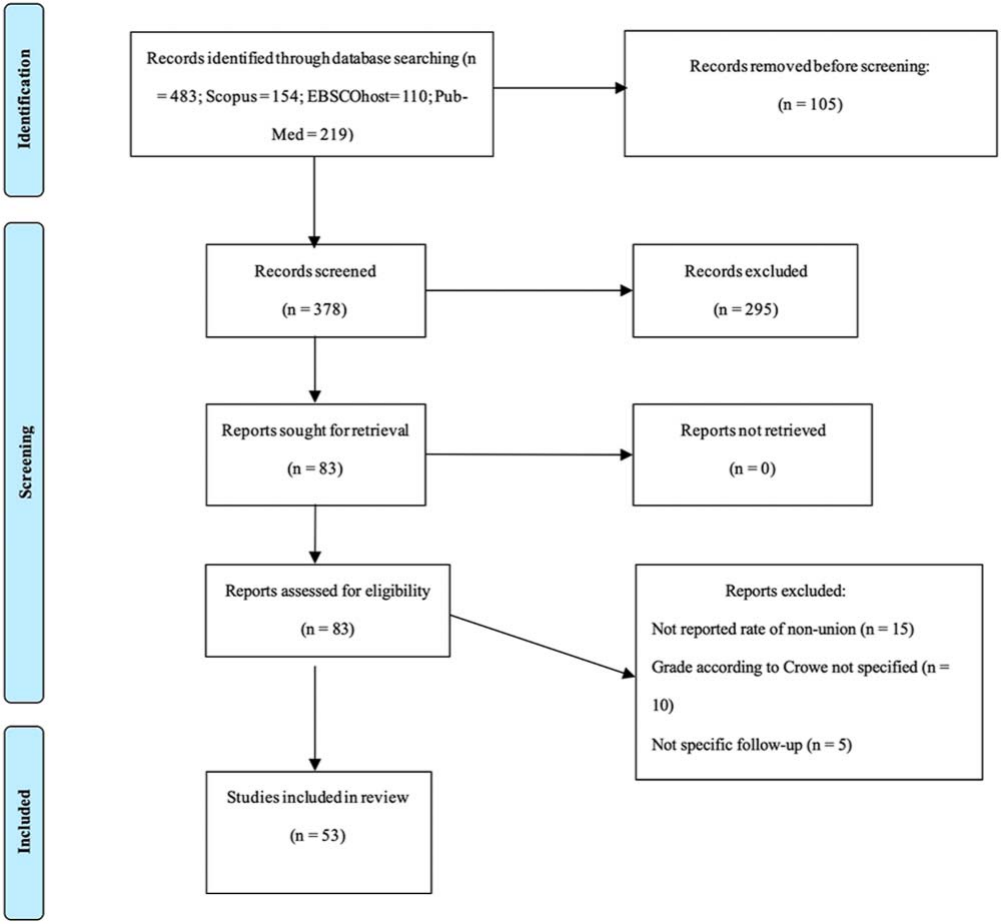
Flow chart of study selection: this flowchart shows the search strategy and the number of identified studies on femoral shortening osteotomy techniques associated with THA in patients with DDH, following the Preferred Reporting Items for Systematic Reviews and Meta-analyses guidelines (94).

A total of 483 studies were identified through the initial search, and 53 studies (9, 12, 14, 18, 20, 22, 26, 27, 35, 36, 37, 38, 39, 40, 41, 42, 43, 44, 45, 46, 47, 48, 49, 50, 51, 52, 53, 54, 55, 56, 57, 58, 59, 60, 61, 62, 63, 64, 65, 66, 67, 68, 69, 70, 71, 72, 73, 74, 75, 76, 77, 78, 79) were included after applying the inclusion and exclusion criteria. All but six of the studies were retrospective case series (level of evidence: IV). The remaining six (46, 52, 55, 60, 71, 76) were retrospective comparative studies (level of evidence: III) (Fig. 1). The mean methodological index for non-randomized studies score was 13.57 (range: 12–14) for non-comparative studies and 19.83 (range: 19–20) for the six comparative studies (Supplementary Material, Table S1).

### Study characteristics

A total of 1,571 patients (1,925 hips) with a mean age of 47.26 (range: 16–85) years were involved. The mean follow-up duration was 6.78 (range: 1–13.7) years. The average length of the shortening osteotomy segment was 3.47 (range: 2–6.65) cm. The type of osteotomy fixation was reported in 40 out of 53 studies (75.47%). Cerclages were used in 28 studies (70%), a plate with screws or cerclages was used in 10 studies (25%), a tension-band wiring was used in 2 studies (5%), and no internal fixation was used in one study (2.5%). Table 1 reports the information about the type of osteotomy, type of stem, and type of cementation of the included studies.

**Table 1:** General characteristics. Data are presented as *n* (%).

Study characteristics	Studies, *n* (%)	Hips, *n* (%)
Total *n*	53	1,925
Year of publication		
1999–2005	2 (3.77)	
2006–2011	10 (18.86)	
2012–2017	18 (33.96)	
2018–2023	23 (43.41)	
Type of osteotomy		
Transverse	39 (73.58)	1,480 (76.88)
Oblique	8 (15.09)	195 (10.13)
Step-cut	6 (11.32)	94 (4.88)
Z-shaped	3 (5.66)	59 (3.06)
Double chevron	2 (3.77)	37 (1.92)
V-shaped	1 (1.88)	26 (1.35)
Distal transverse	1 (1.88)	12 (0.62)
Cementation of stem		
Cemented	8 (15.09)	133 (6.91)
Uncemented	49 (92.45)	1,792 (93.10)
Type of stem		
S-ROM	16 (30.18)	438 (22.75)
Wagner	6 (11.33)	267 (13.87)
Synergy	5 (9.43)^†^	45 (2.34)
SL-Plus*		182 (9.45)
Solution	4 (7.55)	14 (0.72)
SecurFit	3 (5.66)	63 (3.27)
CDH		49 (2.59)
BiContact	2 (3.77)^‡^	11 (0.57)
Anatomic AB		6 (0.31)
Summit*		4 (0.20)
Omnifit		76 (3.94)
Exeter		31 (1.61)
Kyocera		40 (2.07)
Modulus	1 (1.88)^§^	13 (0.67)
Bantam		7 (0.36)
Prodigy		4 (0.20)
AML		4 (0.20)
Spectron		1 (0.05)
Ribbed		11 (0.57)
Corail		17 (0.88)
FMT		14 (0.72)
Helios		68 (3.53)
Integral		31 (1.61)
Landanger		41 (2.13)
Lubinus		14 (0.72)
Restoration HA		9 (0.46)
Consensus		3 (0.15)
Echelon		8 (0.41)
CSR Japan		5 (0.25)
Cannulock		1 (0.05)
H3		4 (0.20)
Lord		2 (0.10)
K-Max		4 (0.20)
Not specified	11 (20.75)	359 (18.65)

* One study did not specify in how many hips the stem was used.

^†^ Relates to Synergy and SL-Plus.

^‡^ Relates to BiContact, Anatomic AB, Summit, Omnifit, Exeter, and Kyocera.

^§^ Relates to Modulus, Bantam, Prodigy, AML, Spectron, Ribbed, Corail, FMT, Helios, Integral, Landanger, Lubinus, Restoration HA, Consensus, Echelon, CSR Japan, Cannulock, H3, Lord, and K-Max.

### Primary outcome

#### Non-union rate

**Figure 2:**
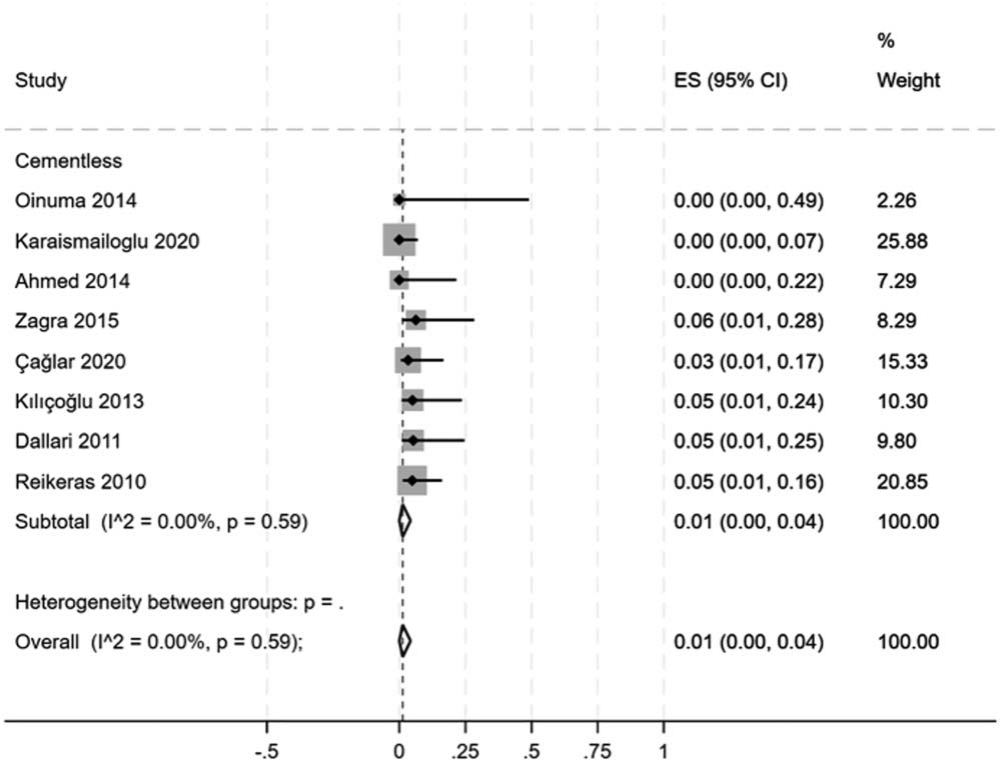
Forest plot showing the prevalence of non-union in oblique osteotomy.

**Figure 3:**
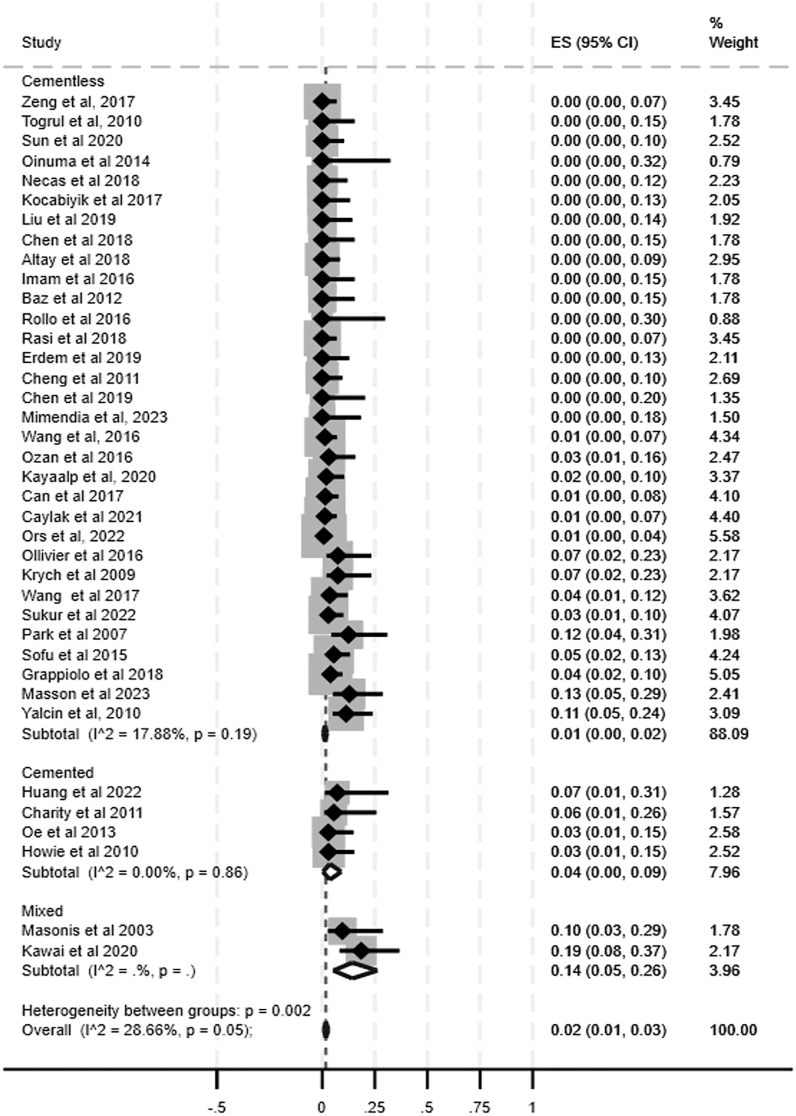
Forest plot showing the prevalence of non-union in transverse osteotomies.

The non-union rate was reported by 53 studies (*n* = 1,925 hips). Considering all osteotomy types, 52/1,925 hips (2.7%) developed non-union. Stratifying by each osteotomy type, proportional meta-analysis showed a non-union prevalence ranging from 0% for step-cut osteotomies (95% CI: 0–0%, *I*^2^ = 0.00%) and Z-osteotomies (95% CI: 0–3%, *I*^2^ = 0.00%) to 2% for transverse osteotomies (95% CI: 1–3%, *I*^2^ = 28.88%) (Supplementary materials, Figs S1, S2, S3, S4). Instead, for the second most commonly used type of osteotomy, oblique osteotomy, among the studies included in the review, the proportional meta-analysis showed a non-union prevalence of 1% (95% CI: 1–4%, *I*^2^ = 0.00%) (Fig. 2). Considering the use of cemented and cementless stems, a higher prevalence of 4% was found for the cemented stems in transverse osteotomy ones (95% CI: 0–9%, *I*^2^ = 0.00%) (Fig. 3). No meta-analysis was performed to investigate the non-union rate for V-shaped (0%, 0/26 hips), distal transverse (0%, 0/12 hips), and mixed (i.e., transverse or step-cut osteotomies) (0%, 0/22 hips) types because they were included in a single study.

#### Revision for non-union

**Figure 4:**
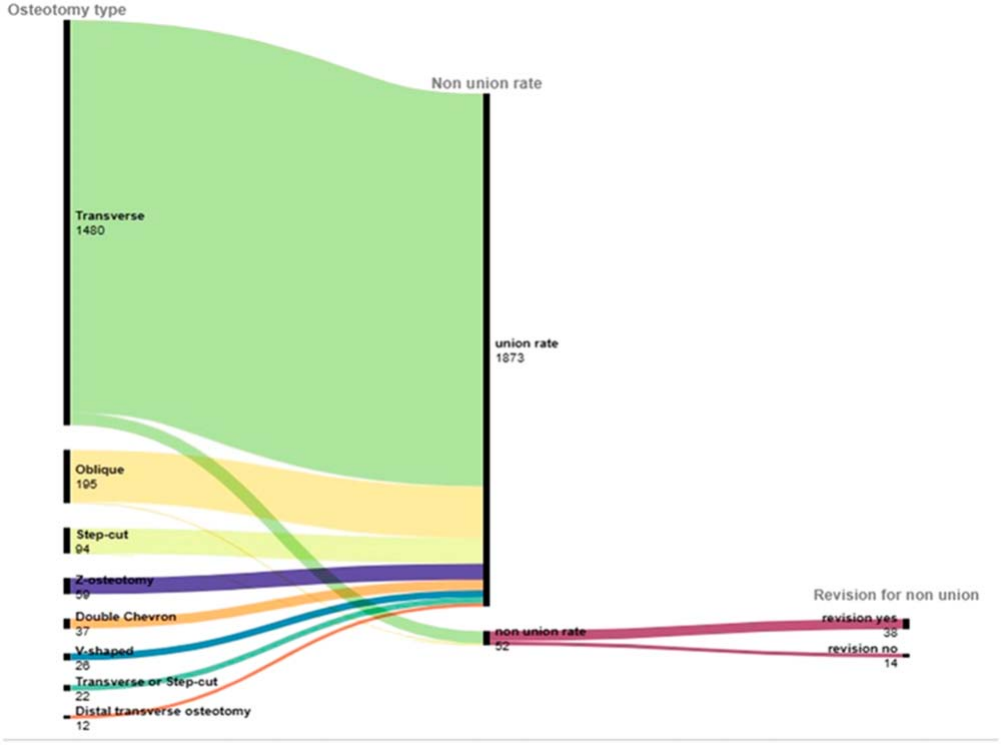
Alluvial plots showing non-union and revision for non-union.

Overall, 52 studies (*n* = 1,900 hips) reported revision for non-union (Fig. 4). Out of the 52 hips that developed non-union, 38 (73%) underwent revision surgery, while the remaining cases were either asymptomatic or patients declined revision surgery. Stratifying by each osteotomy type, transverse osteotomy showed the highest revision rate (33/45; 73.33%), while oblique osteotomy had the lowest (4/6; 66.67%). One study reported one revision for one non-union in step-cut osteotomy (75).

### Secondary outcomes

**Figure 5:**
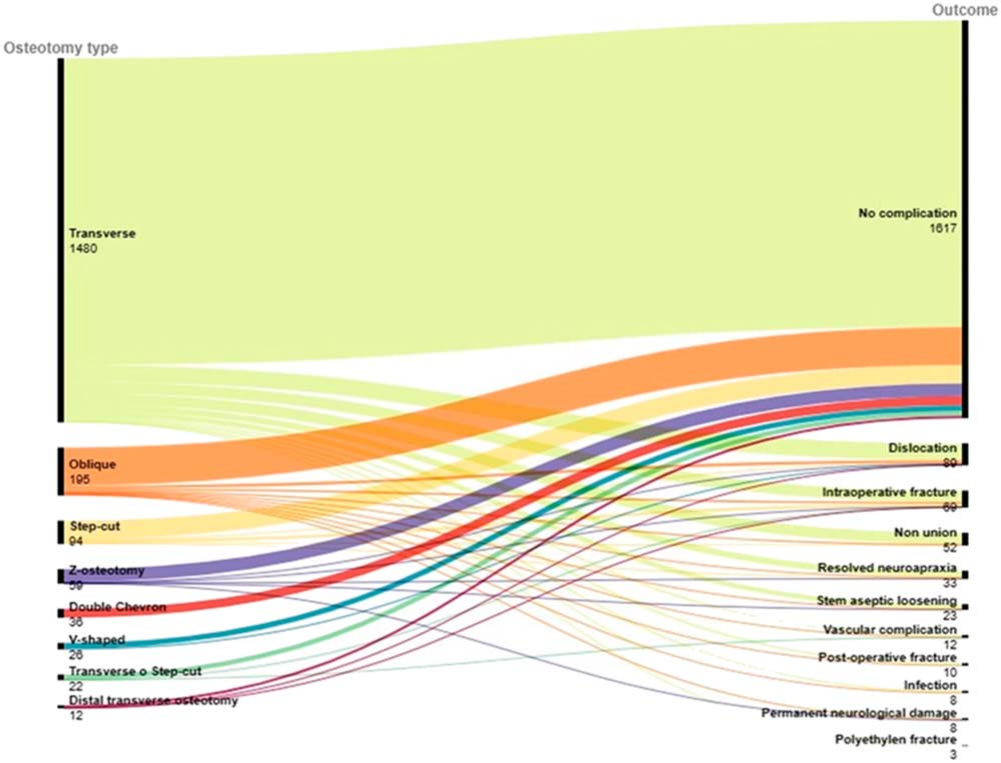
Alluvial plots showing complications in each osteotomy type.

Overall, 383 complications were found. Figure 5 reports alluvial plots showing all complications by osteotomy type, which are stratified by each complication in Supplementary Materials (Figs S5, S6, S7).

#### Dislocation

Totally, 46 studies (*n* = 1,747 hips) reported dislocation. Considering all osteotomy types, 90/1,747 hips (5.15%) developed dislocation. Stratifying by each osteotomy type, V-shaped osteotomies showed the highest dislocation rate (3/26; 11.54%), while double chevron and mixed showed the lowest (0%).

In total, 21 studies (*n* = 51 dislocations) reported revision for dislocation. Considering all osteotomy types, 26/51 dislocated hips (50.98%) underwent revision, while the remaining cases were managed with closed reduction followed by conservative treatment, such as bracing and activity modification. Stratifying by each osteotomy type, transverse osteotomies showed the highest revision rate (21/39; 53.85%), while Z osteotomies showed the lowest (0/3; 0%).

#### Stem aseptic loosening

Stem aseptic loosening was reported by 29 studies (*n* = 1,187 hips). Considering all osteotomy types, 24/1,187 (2.02%) developed stem aseptic loosening. Stratifying by each osteotomy type, Z osteotomy showed the highest step aseptic loosening (1/14; 7.14%), while step-cut (0/2; 0%) and V-shaped osteotomies showed the lowest (0/26; 0%).

Revision for stem aseptic loosening was reported by 14 studies (*n* = 21 stem aseptic loosening). Considering all osteotomy types, 17/21 (80.95%) stem aseptic loosening underwent revision. Stratifying by each osteotomy type, oblique osteotomy showed the highest revision rate (2 revisions on 2 stem aseptic loosening; 100%), while mixed and Z osteotomies showed the lowest (0/1; 0%).

#### Infection

Totally, 24 studies (*n* = 989 hips) reported periprosthetic joint infections. Considering all osteotomy types, 9/989 hips (0.91%) developed infection. Stratifying by each osteotomy type, oblique osteotomy showed the highest infection rate (1/38; 2.63%) followed by transverse osteotomy (8/898, 0.89%), while other osteotomies had the lowest infection rate (0%), except for distal osteotomy and V-shaped osteotomy, which did not report the outcome.

Seven studies (*n* = 6 infections) reported revision for septic loosening. Considering all osteotomy types, 6/6 infected hips (100%) underwent revision in oblique (*n* = 1) and transverse (*n* = 5) osteotomies.

In addition, only one study reported a single case of osteotomy site infection out of 33 hips treated with transverse osteotomy. This patient underwent implant revision surgery (20).

#### Other complications

Other complications were postoperative fracture (8/373 fractures; 6 revisions), polyethylene fracture (3/192 fractures; 3 revisions), and stem fracture (2/89 fracture; 2 revisions). Both reported stem fractures occurred in cases of non-union at the osteotomy site.

Of the other complications reported in the included studies, heterotopic ossification occurred in 72/850 hips (8.47%), intraoperative femoral fracture occurred in 69/1,057 hips (6.53%), resolved neuropraxia occurred in 33/687 hips (4.80%), neurological permanent damage occurred in 8/1,016 cases (0.79%), consisting of persistent palsy due to sciatic nerve injury, and vascular complications occurred in 12/812 hips (1.48%), including deep vein thrombosis and pulmonary embolism.

#### Clinical outcomes

Preoperative and postoperative Trendelenburg and Harris Hip Score were recorded and are provided in Tables 2 and 3. Overall, step-cut osteotomies showed the highest improvement in the Harris Hip Score, with a mean increase of 51.9 points after surgery. Z osteotomies showed the highest rate of Trendelenburg sign remission after surgery (89.65% of patients).

**Table 2:** Report of Harris Hip Scores (95).

	Number of		Harris Hip Score		
	Studies	Hips	Pre-op	Post-op	IMP
Transverse osteotomy	25	1,038	40.35	86.54	46.19
Oblique osteotomy	4	101	43.52	84.97	41.45
Double chevron	2	37	48	87	39
Step-cut	1	35	40.6	92.5	51.9
Z osteotomy	1	37	41.3	84.7	43.4
Distal transverse osteotomy	1	12	41	85	44

IMP, improvement; op, operative.

**Table 3:** Report of presence of Trendelenburg sign (96).

	Number of		Presence of Trendelenburg		
	Studies	Hips	Pre-op	Post-op	REM
Transverse osteotomy	13	433	352	88	75%
Oblique osteotomy	3	85	69	18	73.91%
Double chevron	2	37	28	12	57.14%
Step-cut	2	68	56	10	82.14%
Z osteotomy	1	37	29	3	89.65%
Distal transverse osteotomy	1	12	12	5	58.33%

REM, remission; op, operative.

## Discussion

Femoral shortening osteotomy is a promising surgical intervention for patients with sequelae of high hip dysplasia who need a THA. It offers potential benefits, such as preserving limb length, reducing joint stiffness, and preventing neurovascular damage. However, it is considered a complex procedure with a high risk of complications. Various techniques are employed, and their relative safety remains unclear. Our systematic review analyzed 53 retrospective studies, evaluating clinical outcomes, complications, and revision rates in different techniques of femoral shortening osteotomy.

The results showed an overall rate of non-union of 2.7%, with similar rates among the different techniques. The rate of non-union requiring implant revision was higher for non-union cases of transverse osteotomy (73.33%) than that of oblique osteotomy (66.67%). Li *et al.* (80), in a meta-analysis of 2014 in which they included 37 studies with 795 hips, found an overall rate of non-union at the osteotomy site of 3.79 and 4.14% considering transverse osteotomy. Although slightly higher, these results are similar to those in our study. In other studies, the non-union rate is higher, ranging from 8 to 29% (5, 47, 74).

Most of the studies in our review reported using different surgical techniques, different implant designs, and whether the stem was cemented or uncemented. However, because not all studies reported this information, it was impossible to assess primary and secondary outcomes based on the type of implant design.

In contrast, as all studies reported, assessing the outcomes considering stem cementation was possible. For the transverse osteotomy, the rate of non-union for cemented and uncemented stems was 4 and 1%, respectively. The higher rate of non-union in cemented stems could be due to the possible cement extrusion at the osteotomy site, resulting in non-union (42, 81). An increase in osteotomy healing time has also been seen with cemented stems at about 8 months compared with 3–6 months of cementless stems. Inoue *et al.* (55), in a retrospective study comparing 13 THA with cemented stems and 13 THA with uncemented stems, showed no significant differences at follow-up. Generally, patients undergoing hip arthroplasty surgery for DDH are relatively young, so the tendency is to use uncemented stems. However, most uncemented stems have proximal grip, which cannot guarantee total stability at the subtrochanteric osteotomy. For this reason, modular stems with some distal fixation are most frequently used in this type of surgery (18, 72, 82, 83, 84, 85), and in our review, the most used stem was the S-ROM stem (22.75% of cases). This modular stem is supposed to provide both proximal and distal grip, ensuring a greater fit and thus the stability necessary for the healing of the osteotomy. At the same time, this exposes a greater risk of intraoperative fractures. In addition, modular stems allow for a more accurate adjustment of femoral anteversion (80). The second most used type of stem was a conical stem (Wagner, Echelon, and Cannulock, for a total of 276 hips, 14.34% of cases) aimed for distal fixation and with the possibility of managing the neck anteversion more easily.

Furthermore, the femoral stem design may contribute to non-union. Conical and modular stems were observed to have better outcomes than rectangular stems (50, 53, 55). As mentioned above, it was not easy in our study to extrapolate precise data related to non-union, stem loosening, and specific stem types, so we cannot discuss this more deeply.

The type of osteotomy fixation was reported in 75.47% of studies, and a device was used in 97.5% of studies. However, it was impossible to accurately assess the rate of non-union or other outcomes regarding the type of device for fixation used because, in most studies, it was not specified in how many patients it was used or not.

The overall dislocation rate was 5.15%, with the highest rate for V-shaped and transverse osteotomies (11.54 and 4.99%, respectively). These results were like those found by Li *et al.*, with a total dislocation rate of 5.88%. Similarly, the rest of the literature reports a frequency of dislocation after the first implantation prosthesis in the DDH between 2.9 and 11% (81), and it is higher if risk factors such as age, sex, smaller femoral head diameter, surgical approach, and surgeon experience are present (86). Underlying the increased risk of dislocation seem to be more decisive factors related to intrinsic anatomic-functional alterations and factors related to surgical technique (87), such as hyposthenia of the abductors, lack of adequate capsular containment, and the frequent use of cups and heads with smaller diameters. Although dislocation is a multifactorial event influenced by various factors, it is possible that the type of osteotomy itself may contribute to the risk of dislocation. In particular, we hypothesize that the higher dislocation rate observed in V-shaped and transverse osteotomies could be attributed to the technical challenges associated with achieving adequate trochanteric rotational control, potentially leading to increased implant instability. Since the included studies did not consistently report data on factors such as abductor function, offset restoration, or precise component positioning, and considering that the number of cases analyzed, particularly for V-shaped osteotomies, was limited, it is not possible to draw definitive conclusions.

Considering all osteotomy types, 0.91% developed infection. There are no apparent differences between the different types of osteotomies, as also evidenced by Li *et al.* Infections may be more frequent if risk factors related to patient predisposition and comorbidities and intra- and postoperative risk factors are present, as found in studies assessing the risk of infection in primary implant prosthetic surgeries (80). Compared with transverse osteotomy, the other osteotomies considered in our study seem to require more surgical time because of their complexity (28, 84, 85), and several studies have shown that prolonged operative time increases the risk of deep infection after THA (88, 89, 90). Therefore, one might hypothesize that transverse osteotomy might be associated with a lower infection rate than other types of osteotomies. However, we found similar infection rates among the different types of osteotomies.

Previous studies reported neurological damage incidence from 4–5% to 11.3% (37, 91). The etiology of nerve damage is debated. Lengthening beyond 3–4 cm is thought to increase the risk of neurological damage (6, 7). Some authors say that it is more likely due to direct intraoperative damage rather than limb lengthening (40, 91). Typically, in most cases, the symptoms of neurological damage resolve within 6 months (91). These observations were confirmed in our analysis. Only 0.79% of neurological deficits were permanent and required subsequent procedures to improve limb function. At the same time, resolved neuropraxia occurred in 4.80%.

In our systematic review, the incidence of intraoperative femoral fractures was 6.53%, which agrees with the expected rate. Fixation with cerclages was sufficient in most cases. The literature describes a frequency of 5–22% (5, 10, 83, 92). The incidence is 14% with the use of rectangular stems (93). In contrast, using conical or modular stems seems to reduce the incidence of intraoperative fractures (40).

From a clinical perspective, our study aimed to evaluate whether different types of femoral shortening osteotomies impacted the Harris Hip Score (HHS) and the frequency of the Trendelenburg sign. Our analysis showed that improvement in HHS and resolution of the Trendelenburg sign were observed across all osteotomy types, suggesting that femoral shortening osteotomies in THA contribute positively to functional recovery, in agreement with the significant improvement in expected pain reduction (40, 80). However, we fully acknowledge that multiple factors, including implant design, surgical technique, and postoperative rehabilitation, influence the improvement in HHS and the resolution of the Trendelenburg sign. Therefore, the type of osteotomy alone cannot be considered the sole determinant of functional outcomes. In addition, the included studies did not always provide detailed data on other contributing factors, limiting our ability to establish a direct causal relationship.

This study has several limitations that should be acknowledged at the review and study levels.

At the review level, only the primary outcome was included in the proportional meta-analysis, while descriptive statistics were used for secondary outcomes. This limits the statistical power of our findings and makes direct comparisons between osteotomy types more challenging. However, by performing a proportional meta-analysis, we could do an indirect comparison by assessing whether CIs overlapped. In addition, only retrospective studies were included, with only six comparative ones and without a high level of evidence, based on a limited number of cases and characterized by variability in study protocols. It is possible that despite a thorough search, no relevant studies on the topic were identified.

At the study level, we found a high imbalance in the representation of osteotomy types, with transverse osteotomy accounting for 78% of hips. This overrepresentation may reduce the significance of comparisons between osteotomy techniques. In addition, many studies are from non-European countries, with a higher overall number of cases due to the diversity of newborn screening applications and the dissimilarity of follow-up patients with DDH. Furthermore, non-English-language papers were excluded, and most studies were conducted in high-volume centers, making replication of these results in lower-volume institutions difficult.

Another significant limitation is the heterogeneity among included studies, particularly in patient populations, surgical techniques, and outcome reporting. This variability limits our ability to draw definitive conclusions regarding the superiority of one femoral shortening osteotomy technique over another. Although femoral shortening osteotomies are often described as technically demanding procedures with a potentially high complication rate, it should be emphasized that outcomes may differ substantially across surgical contexts. In high-volume referral centers, where these procedures are performed by experienced surgeons – particularly in the case of transverse and oblique shortening osteotomies – they can be safe and reproducible techniques. Therefore, the complication rates reported in this review, derived from studies encompassing a wide range of surgical settings and expertise levels, may not fully reflect the results achievable in specialized institutions.

Moreover, not all studies reported data on implant characteristics or osteotomy fixation methods, leaving two clinically important questions unanswered: Does the type of implant influence complication rates and outcomes? Does the type of osteotomy fixation affect healing and stability? These aspects play a crucial role in the success of femoral shortening osteotomies, yet the available data did not allow a precise evaluation of their impact.

Extracting precise data on non-union and stem loosening about specific stem designs was challenging due to inconsistent reporting across studies. One study’s observation of a high non-union rate in the cemented stem group (30%) (67) suggests a possible advantage of uncemented stems. However, since this finding was derived from a single study, it may not be generalizable, and no definitive conclusions can be drawn.

However, this is one of the few reviews comparing different osteotomy techniques for femoral shortening in patients with DDH undergoing THA surgery. An updated systematic search and literature review was performed, filtered by inclusion and exclusion criteria. This systemic review and proportional meta-analysis provide the most recent information on this topic.

## Conclusion

Our systemic review and proportional meta-analysis showed that transverse osteotomy is the most commonly used technique in the literature and that there is an overlapping prevalence in non-union rates among the different osteotomy techniques. Comparable rates were demonstrated between the use of any technique regarding neurological damage, Harris Hip Score, presence of Trendelenburg, and need for revision. These results showed that all types of osteotomies have an overall low risk of complications, with V-shaped osteotomies displaying the highest prevalence of dislocation (11.54%) among all complications.

Although we followed a rigorous methodology, the limitations in the analyzed studies prevented us from reaching unequivocal conclusions. Further studies, including randomized controlled trials, will be needed to confirm and update our results.

## Supplementary materials



## ICMJE Statement of Interest

Each author certifies that there are no funding or commercial associations (consultancies, stock ownership, equity interest, patent/licensing arrangements, etc.) that might pose a conflict of interest in connection with the submitted article related to the author or any immediate family members.

## Funding Statement

The work was supported and funded by the Italian Ministry of Health - “Ricerca Corrente”. The APC was funded by Italian Ministry of Health - “Ricerca Corrente”.
