# Supplementary material for: What is the femoral shortening osteotomy in THA for congenital high hip dislocation with the lowest complication rate? A systematic review
Source: EFORT Open Rev. 2026 Feb 4;11(2):107–18. doi: 10.1530/EOR-2024-0146 (PMC12881905; doi:10.1530/EOR-2024-0146)
Supplement: Supplementary file 1 [file supplementary_materials.pdf]

SUPPLEMENTARY MATERIALS

What is the femoral shortening osteotomy in THA for congenital high hip dislocation with the lowest complication rate? A systematic review

Appendix 1

Search strategy used in Pubmed

("congenital"[MeSH Subheading] OR "congenital"[All Fields] OR "congenitally"[All Fields] OR ("developmental"[All Fields] OR "developmentally"[All Fields])) AND ("hip"[MeSH Terms] OR "hip"[All Fields]) AND ("dysplasia"[All Fields] OR "dysplasias"[All Fields] OR ("dislocate"[All Fields] OR "dislocated"[All Fields] OR "dislocates"[All Fields] OR "dislocating"[All Fields] OR "dislocator"[All Fields] OR "dislocators"[All Fields] OR "joint dislocations"[MeSH Terms] OR ("joint"[All Fields] AND "dislocations"[All Fields]) OR "joint dislocations"[All Fields] OR "dislocation"[All Fields] OR "dislocations"[All Fields]) OR ("dislocate"[All Fields] OR "dislocated"[All Fields] OR "dislocates"[All Fields] OR "dislocating"[All Fields] OR "dislocator"[All Fields] OR "dislocators"[All Fields] OR "joint dislocations"[MeSH Terms] OR ("joint"[All Fields] AND "dislocations"[All Fields]) OR "joint dislocations"[All Fields] OR "dislocation"[All Fields] OR "dislocations"[All Fields])) AND ("subtrochanteric"[All Fields] AND ("osteotomie"[All Fields] OR "osteotomied"[All Fields] OR "osteotomy"[MeSH Terms] OR "osteotomy"[All Fields] OR "osteotomies"[All Fields] OR ("osteotomie"[All Fields] OR "osteotomied"[All Fields] OR "osteotomy"[MeSH Terms] OR "osteotomy"[All Fields] OR "osteotomies"[All Fields]))).

Table S1: Results of the Methodological Index for Non-randomized Studies (MINORS) Score.

Author	stated aim	Inclusion of patients	collection of data	Endpoints appropriate to the aim	Unbiased assessment of the study endpoint	Follow-up	Loss to follow up less than 5%	Prospective calculation of the study size	Control group	Contemporary groups	Baseline equivalence of groups	statistical analyses
**Zeng et al. 2017 (1)**	2	2	2	2	0	2	2	0	0	0	0	2
**Zagra et al. 2015 (2)**	2	2	2	2	0	2	2	0	0	0	0	2
**Yalcin et al 2010 (3)**	2	2	2	2	0	2	2	0	0	0	0	2
**Wang et al 2016 (4)**	2	2	2	2	0	2	2	0	0	0	0	2
**BİÇİCİ et al 2021 (5)**	2	2	2	2	0	2	2	0	0	0	0	2
**Togrul et al 2010 (6)**	2	2	2	2	0	2	2	0	0	0	0	0
**Takao et al 2011 (7)**	2	2	2	2	0	2	2	0	0	0	0	2
**Sun et al 2020 (8)**	2	2	2	2	0	2	2	0	0	0	0	2

**Sofu et al 2015 (9)**	2	2	2	2	0	2	2	0	0	0	0	1
**Park et al 2007 (10)**	2	2	2	2	0	2	2	0	0	0	0	0
**Huang et al 2022 (11)**	2	2	2	2	0	2	2	0	0	0	0	2
**Ollivier et al 2016 (12)**	2	2	2	2	0	2	2	0	0	0	0	2
**Oinuma et al 2014 (13)**	2	2	2	2	0	2	2	0	0	0	0	2
**Necas et al 2018 (14)**	2	2	2	2	0	2	2	0	0	0	0	2
**Masonis et al 2003 (15)**	2	2	2	2	0	2	2	0	0	0	0	0
**Ozan et al 2016 (16)**	2	2	2	2	0	2	2	0	0	0	0	0
**Liu et al 2019 (17)**	2	2	2	2	0	2	2	0	0	0	0	2
**Li et al 2016 (18)**	2	2	2	2	0	2	2	0	0	0	0	1
**Krych et al 2009 (19)**	2	2	2	2	0	2	2	0	0	0	0	2
**Kayaalp et al. 2020 (20)**	2	2	2	2	0	2	2	0	0	0	0	2
**Çağlar et al 2020 (21)**	2	2	2	2	0	2	2	0	0	0	0	2
**Chen et al 2018 (22)**	2	2	2	2	0	2	2	0	0	0	0	1
**Altay et al 2018 (23)**	2	2	2	2	0	2	2	0	0	0	0	2
**Guo et al 2015 (24)**	2	2	2	2	0	2	2	0	0	0	0	0
**Grappiolo et al 2018 (25)**	2	2	2	2	0	2	2	0	0	0	0	2
**Imam et al 2016 (26)**	2	2	2	2	0	2	2	0	0	0	0	2
**Baz et al 2012 (27)**	2	2	2	2	0	2	2	0	0	0	0	2
**Wang et al 2017 (4)**	2	2	2	2	0	2	2	0	0	0	0	2
**Reikera°s et al 2010 (28)**	2	2	2	2	0	2	2	0	0	0	0	1

**Rollo et al 2016 (29)**	2	2	2	2	0	2	2	0	0	0	0	2
**Rasi et al 2018 (30)**	2	2	2	2	0	2	2	0	0	0	0	0
**Charity et al 2011 (31)**	2	2	2	2	0	2	2	0	0	0	0	2
**Masson et al 2023 (32)**	2	2	2	2	0	2	1	0	0	0	0	2
**Kılıçoğlu et al 2013 (33)**	2	2	2	2	0	2	2	0	0	0	0	2
**Kawai et al 2020 (34)**	2	2	2	2	0	2	2	0	0	0	0	2
**Caylak et al 2021 (35)**	2	2	2	2	0	2	1	0	0	0	0	2
**Erdem et al 2019 (36)**	2	2	2	2	0	2	2	0	0	0	0	2
**Dallari et al 2011 (37)**	2	2	2	2	0	2	2	0	0	0	0	2
**Cheng et al 2011 (38)**	2	2	2	2	0	2	2	0	0	0	0	2
**Oe et al 2013 (39)**	2	2	2	2	0	2	2	0	0	0	0	2
**Howie et al 2010 (40)**	2	2	2	2	0	2	1	0	0	0	0	2
**Akinci et al 2022 (41)**	2	2	2	2	0	2	2	0	0	0	0	2
**Ahmed et al 2014 (42)**	2	2	2	2	0	2	2	0	0	0	0	2
**Charean cholvanich et al. 1999 (43)**	2	2	2	2	0	2	2	0	0	0	0	2
**Sukur et al 2022 (44)**	2	2	2	2	0	2	2	0	0	0	0	2
**Mimendia et al. 2023 (45)**	2	2	2	2	0	2	2	0	0	0	0	2
**Ors et al. 2022 (46)**	2	2	2	2	0	2	2	0	0	0	0	2
**Chen et al 2019 (47)**	2	2	2	2	0	2	2	0	2	2	2	2
**Ravanbod et al 2023 (48)**	2	2	2	2	0	2	2	0	2	2	2	2

**Can et al 2017 (49)**	2	2	2	2	0	2	2	0	2	2	2	2
**Inoue et al 2020 (50)09/01/2026 11:36:00**	2	2	2	2	0	2	2	0	2	2	2	2
**Kocabiyik et al 2017 (51)**	2	2	1	2	0	2	2	0	2	2	2	2
**Karaismailoglu et al 2020 (52)**	2	2	2	2	0	2	2	0	2	2	2	2

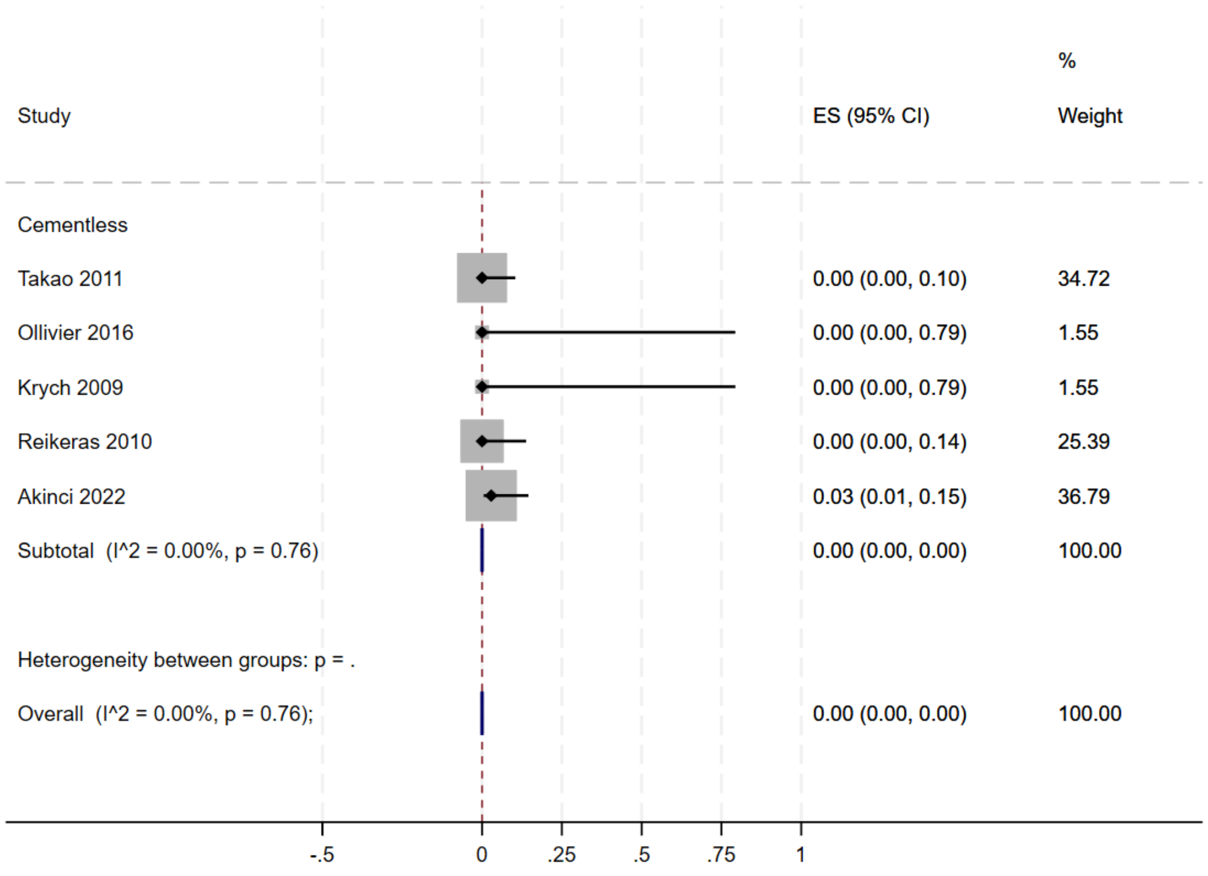

Figure S1. Forest plot showing the prevalence of non-union in Step-cut osteotomies.

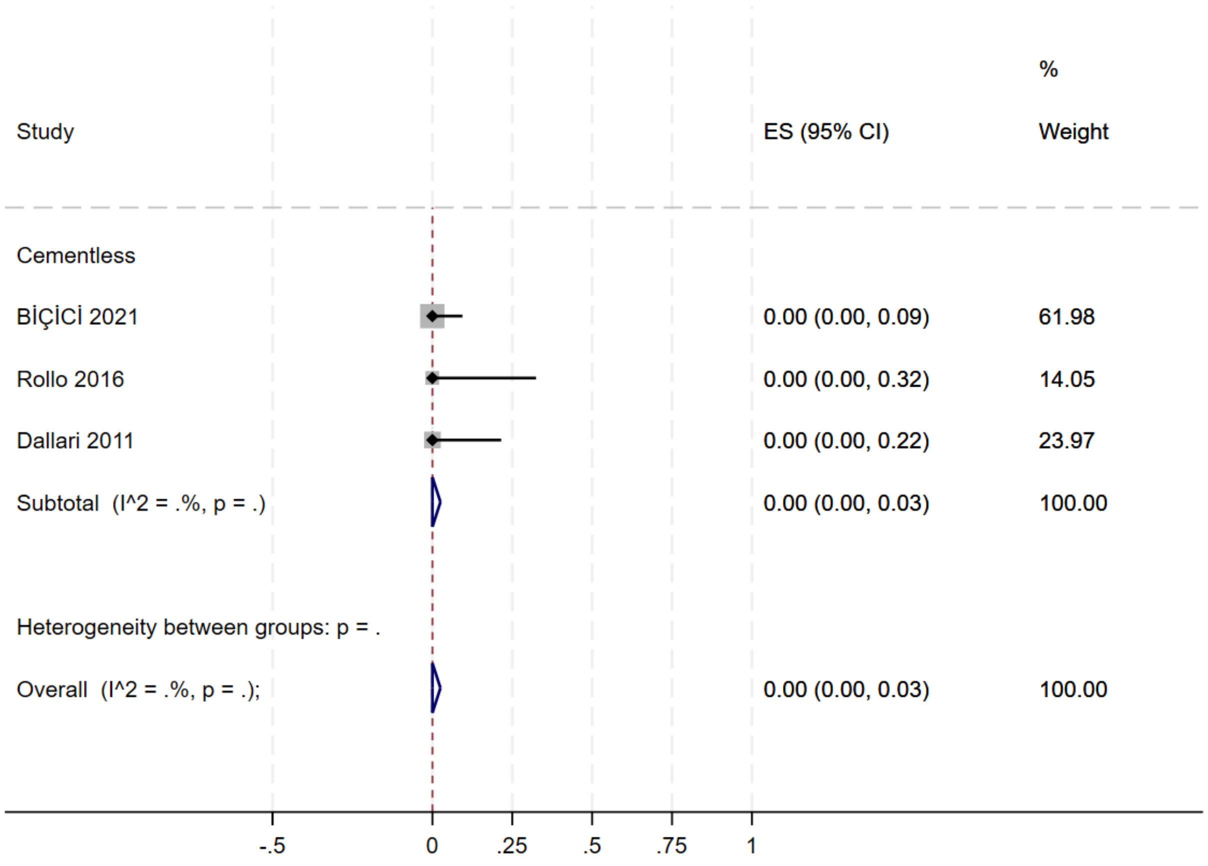

Figure S2. Forest plot showing the prevalence of non-union in Z-osteotomies.

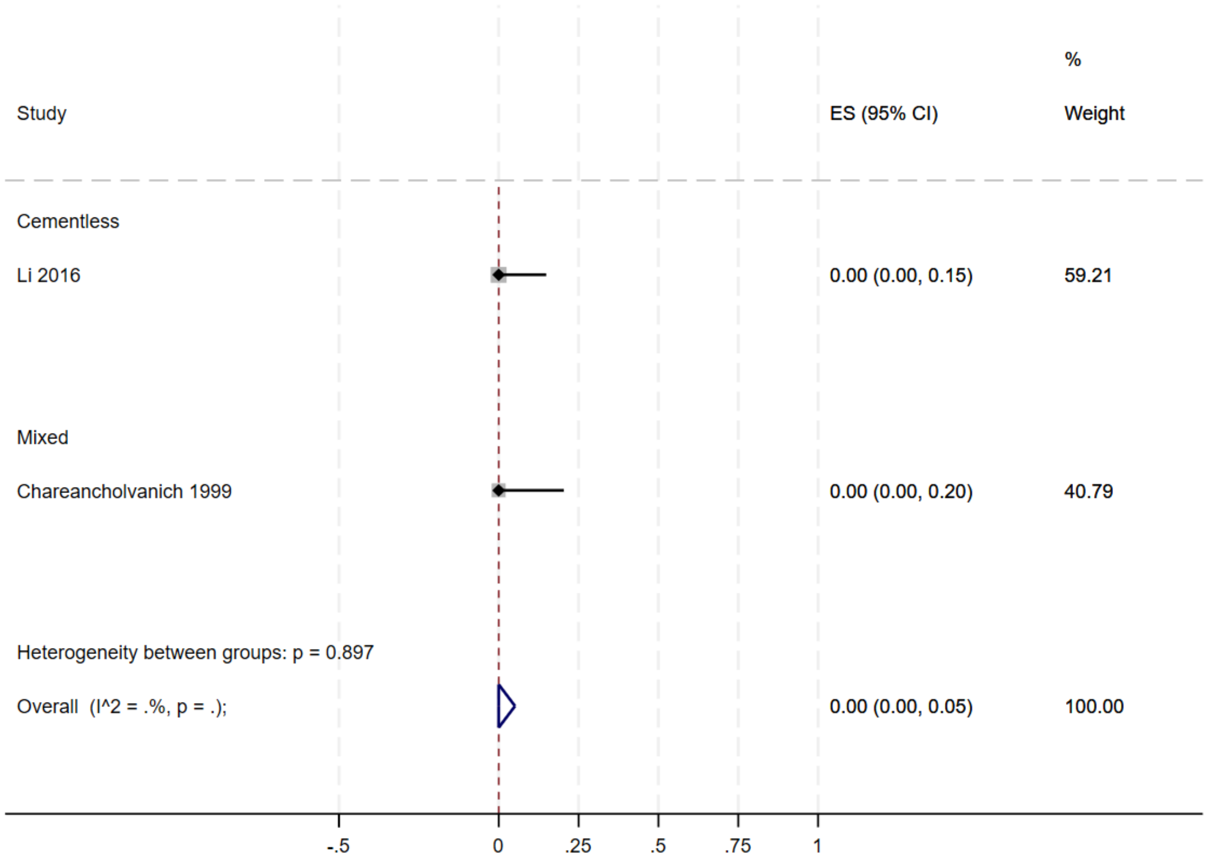

Figure S3. Forest plot showing the prevalence of non-union in Double Chevron osteotomies.

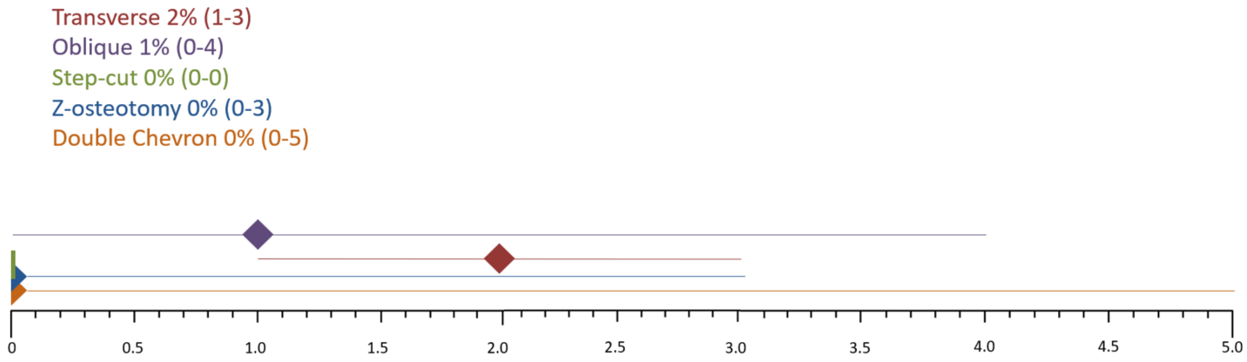

Figure S4. Comparison of different types of osteotomy, showing no significant differences

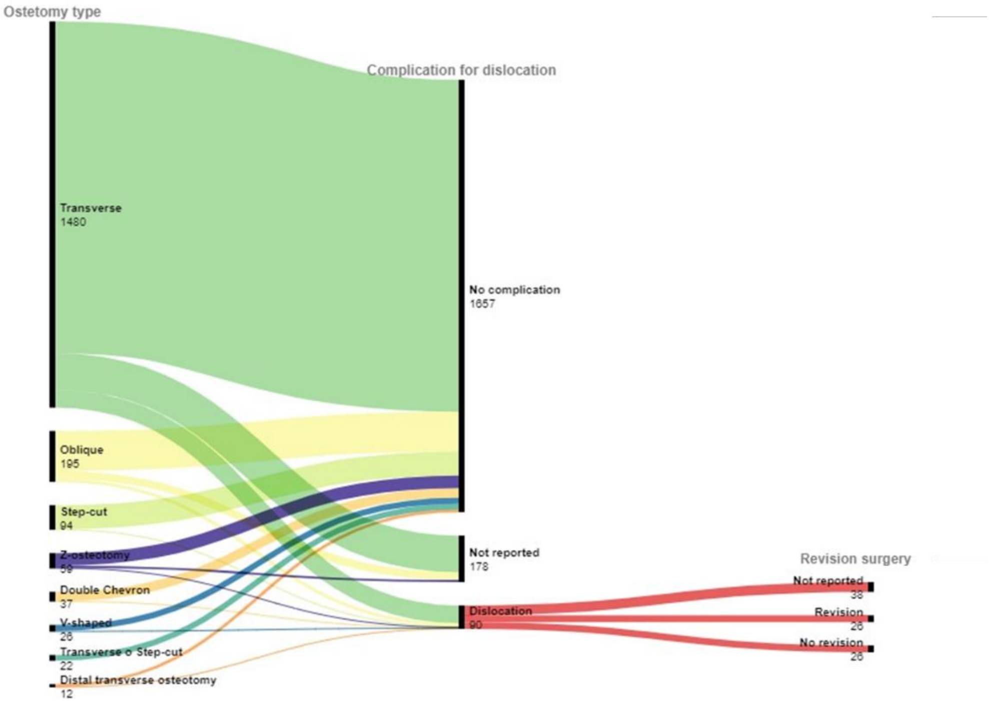

Figure S5. Alluvial plots showing dislocation and revision for dislocation

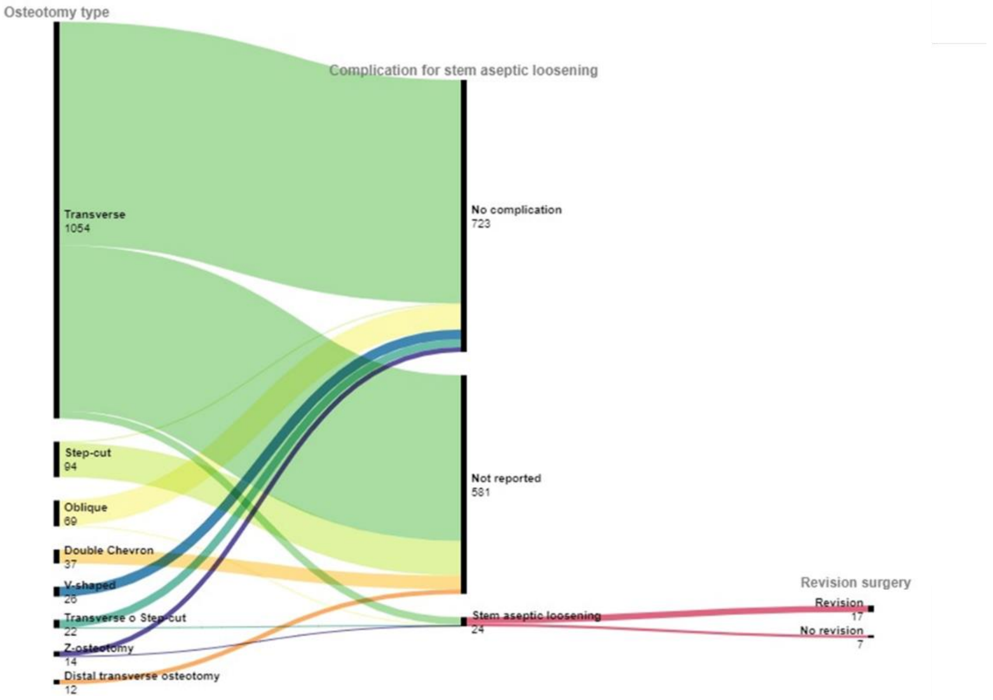

Figure S6. Alluvial plots showing stem aseptic loosening and revision for stem aseptic loosening

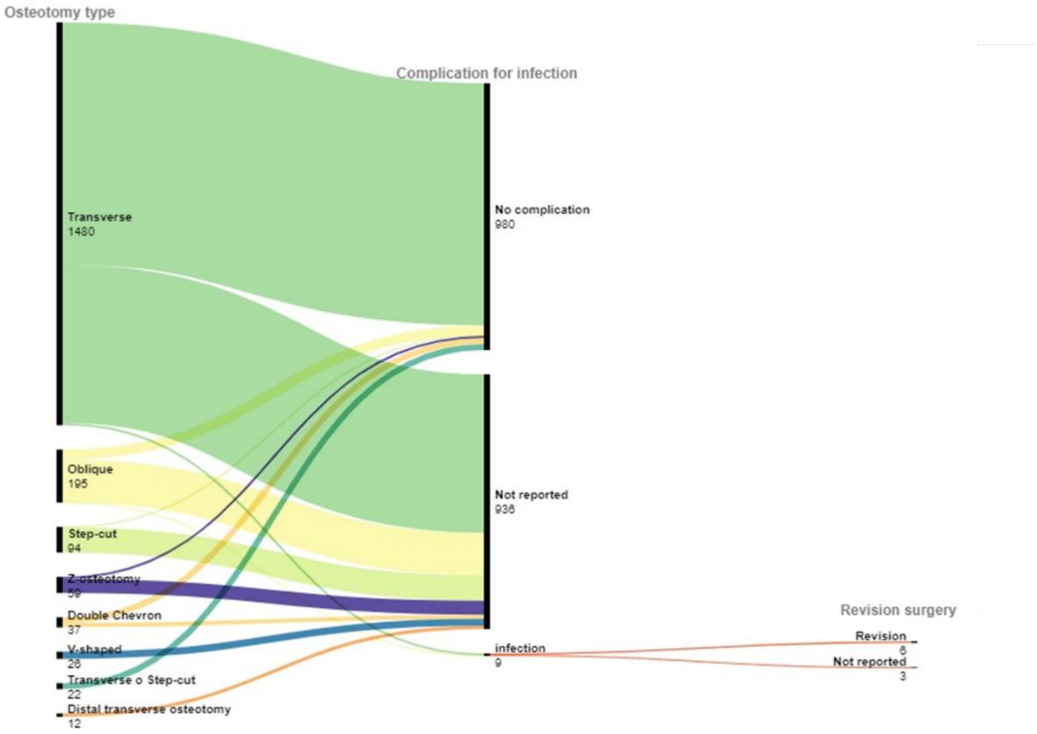

Figure S7. Alluvial plots showing infection and revision for infection

Note: revision for polyetilene fracture (3 fracture and 3 revisions) and post-operative fracture (10 fracture and 10 revisions) are missing in alluvial diagrams

References

1. Zeng WN, Liu JL, Wang FY, Zhang X, Fan HQ, Chen GX, Guo L, Duan XJ, Zhou Q, Yang L. Total hip arthroplasty for patients with Crowe type IV developmental dysplasia of the hip: Ten years results. Int J Surg. 2017 Jun;42:17–21.

2. Zagra L, Bianchi L, Mondini A, Ceroni RG. Oblique femoral shortening osteotomy in total hip arthroplasty for high dislocation in patients with hip dysplasia. Int Orthop. 2015 Sep;39(9):1797–802.

3. Yalcin N, Kilicarslan K, Karatas F, Mutlu T, Yildirim H. Cementless total hip arthroplasty with subtrochanteric transverse shortening osteotomy for severely dysplastic or dislocated hips. Hip Int. 2010;20(1):87–93.

4. Wang D, Li LL, Wang HY, Pei FX, Zhou ZK. Long-Term Results of Cementless Total Hip Arthroplasty With Subtrochanteric Shortening Osteotomy in Crowe Type IV Developmental Dysplasia. J Arthroplasty. 2017 Apr;32(4):1211–9.

5. Biçici V, Bingöl I, Sazak T. Mid-term results of total hip arthroplasty with subtrochanteric Z-osteotomy in Crowe type 3-4 developmental hip dysplasia. Turk J Med Sci. 2021 Aug 30;51(4):1976–83.

6. Togrul E, Ozkan C, Kalaci A, Gülşen M. A new technique of subtrochanteric shortening in total hip replacement for Crowe type 3 to 4 dysplasia of the hip. J Arthroplasty. 2010 Apr;25(3):465–70.

7. Takao M, Ohzono K, Nishii T, Miki H, Nakamura N, Sugano N. Cementless modular total hip arthroplasty with subtrochanteric shortening osteotomy for hips with developmental dysplasia. J Bone Joint Surg Am. 2011 Mar 16;93(6):548–55.

8. Sun C, Zhang Y, Li LT, Ding H, Guo T, Zhao JN. Long-Term Outcomes of Total Hip Arthroplasty With Transverse Subtrochanteric Shortening Osteotomy and Modular Stem in Crowe IV Developmental Dysplasia. J Arthroplasty. 2021 Feb;36(2):630–5.

9. Sofu H, Kockara N, Gursu S, Issin A, Oner A, Sahin V. Transverse Subtrochanteric Shortening Osteotomy During Cementless Total Hip Arthroplasty in Crowe Type-III or IV Developmental Dysplasia. J Arthroplasty. 2015 Jun;30(6):1019–23.

10. Park MS, Kim KH, Jeong WC. Transverse subtrochanteric shortening osteotomy in primary total hip arthroplasty for patients with severe hip developmental dysplasia. J Arthroplasty. 2007 Oct;22(7):1031–6.

11. Huang ZY, Ling J, Zeng ZM, Di ZL, Zhang JH, Tao K. Mid-Term Outcomes of Cemented Stem and Subtrochanteric Shortening Derotational Osteotomy in Total Hip Arthroplasty for Crowe IV Developmental Dysplasia. Orthop Surg. 2022 Dec;14(12):3178–86.

12. Ollivier M, Abdel MP, Krych AJ, Trousdale RT, Berry DJ. Long-Term Results of Total Hip Arthroplasty With Shortening Subtrochanteric Osteotomy in Crowe IV Developmental Dysplasia. J Arthroplasty. 2016 Aug;31(8):1756–60.

13. Oinuma K, Tamaki T, Miura Y, Kaneyama R, Shiratsuchi H. Total hip arthroplasty with subtrochanteric shortening osteotomy for Crowe grade 4 dysplasia using the direct anterior approach. J Arthroplasty. 2014 Mar;29(3):626–9.

14. Necas L, Hrubina M, Melisik M, Cibula Z, Chmurny M, Daniel M, Steno B. Cementless hip arthroplasty and transverse shortening femoral osteotomy with the S-ROM stem for Crowe type IV developmental dysplasia. Eur J Orthop Surg Traumatol. 2019 Jul;29(5):1025–33.

15. Masonis JL, Patel JV, Miu A, Bourne RB, McCalden R, Macdonald SJ, Rorabeck CH. Subtrochanteric shortening and derotational osteotomy in primary total hip arthroplasty for patients with severe hip dysplasia: 5-year follow-up. J Arthroplasty. 2003 Apr;18(3 Suppl 1):68–73.

16. Ozan F, Uzun E, Gürbüz K, Koyuncu Ş, Altay T, Kayalı C. Total hip arthroplasty in the developmental dysplasia of the hip using transverse subtrochanteric osteotomy. J Orthop. 2016 Dec;13(4):259–63.

17. Liu T, Wang S, Huang G, Wang W. Treatment of Crowe IV developmental dysplasia of the hip with cementless total hip arthroplasty and shortening subtrochanteric osteotomy. J Int Med Res. 2019 Jul;47(7):3223–33.

18. Li X, Lu Y, Sun J, Lin X, Tang T. Treatment of Crowe Type-IV Hip Dysplasia Using Cementless Total Hip Arthroplasty and Double Chevron Subtrochanteric Shortening Osteotomy: A 5- to 10-Year Follow-Up Study. J Arthroplasty. 2017 Feb;32(2):475–9.

19. Krych AJ, Howard JL, Trousdale RT, Cabanela ME, Berry DJ. Total hip arthroplasty with shortening subtrochanteric osteotomy in Crowe type-IV developmental dysplasia. J Bone Joint Surg Am. 2009 Sep;91(9):2213–21.

20. Kayaalp ME, Can A, Erdogan F, Ozsahin MK, Aydingoz O, Kaynak G. Clinical and Radiological Results of Crowe Type 3 or 4 Dysplasia Patients Operated on With Total Hip Arthroplasty Using a Cementless Rectangular Femoral Component Without Fixating or Grafting the Transverse Osteotomy Site. J Arthroplasty. 2020 Sep;35(9):2537–42.

21. Caglar O, Isik S, Kaymakoglu M, Demirkiran HG, Atilla B, Tokgozoglu M, Yazici M. Sagittal spinal alignment after total hip arthroplasty for neglected high hip dysplasia: does changing the distorted mechanics of the hip normalize spinal alignment? Spine Deform. 2021 Jan;9(1):221–9.

22. Chen M, Gittings DJ, Yang S, Liu X. Total Hip Arthroplasty for Crowe Type IV Developmental Dysplasia of the Hip Using a Titanium Mesh Cup and Subtrochanteric Femoral Osteotomy. Iowa Orthop J. 2018;38:191–5.

23. Altay M, Demirkale İ, Çatma MF, Karaduman M. Results of Crowe Type IV Developmental Dysplasia of Hip Treated by Subtrochantric Osteotomy and Total Hip Arthroplasty. Indian J Orthop. 2018 Aug;52(4):374–9.

24. Guo CY, Liang BW, Sha M, Kang LQ, Wang JZ, Ding ZQ. Cementless arthroplasty with a distal femoral shortening for the treatment of Crowe type IV developmental hip dysplasia. Indian J Orthop. 2015 Aug;49(4):442–6.

25. Grappiolo G, La Camera F, Della Rocca A, Mazziotta G, Santoro G, Loppini M. Total hip arthroplasty with a monoblock conical stem and subtrochanteric transverse shortening osteotomy in Crowe type IV dysplastic hips. Int Orthop. 2019 Jan;43(1):77–83.

26. Imam MA, Fathalla I, Holton J, Nabil M, Kashif F. Cementless Total Hip Replacement for the Management of Severe Developmental Dysplasia of the Hip in the Middle Eastern Population: A Prospective Analysis. Front Surg. 2016;3:31.

27. Baz AB, Senol V, Akalin S, Kose O, Guler F, Turan A. Treatment of high hip dislocation with a cementless stem combined with a shortening osteotomy. Arch Orthop Trauma Surg. 2012 Oct;132(10):1481–6.

28. Reikerås O, Haaland JE, Lereim P. Femoral shortening in total hip arthroplasty for high developmental dysplasia of the hip. Clin Orthop Relat Res. 2010 Jul;468(7):1949–55.

29. Rollo G, Solarino G, Vicenti G, Picca G, Carrozzo M, Moretti B. Subtrochanteric femoral shortening osteotomy combined with cementless total hip replacement for Crowe type IV developmental dysplasia: a retrospective study. J Orthop Traumatol. 2017 Dec;18(4):407–13.

30. Rasi AM, Kazemian G, Khak M, Zarei R. Shortening subtrochanteric osteotomy and cup placement at true acetabulum in total hip arthroplasty of Crowe III-IV developmental dysplasia: results of midterm follow-up. Eur J Orthop Surg Traumatol. 2018 Jul;28(5):923–30.

31. Charity JAF, Tsiridis E, Sheeraz A, Howell JR, Hubble MJW, Timperley AJ, Gie GA. Treatment of Crowe IV high hip dysplasia with total hip replacement using the Exeter stem and shortening derotational subtrochanteric osteotomy. J Bone Joint Surg Br. 2011 Jan;93(1):34–8.

32. Masson JB, Foissey C, Bertani A, Pibarot V, Rongieras F. Transverse subtrochanteric shortening osteotomy with double tension-band fixation during THA for Crowe III-IV developmental dysplasia: 12-year outcomes. Orthop Traumatol Surg Res. 2023 Sep 11;103684.

33. Kiliçoğlu Oİ, Türker M, Akgül T, Yazicioğlu O. Cementless total hip arthroplasty with modified oblique femoral shortening osteotomy in Crowe type IV congenital hip dislocation. J Arthroplasty. 2013 Jan;28(1):117–25.

34. Kawai T, Goto K, Kuroda Y, Matsuda S. Total Hip Arthroplasty Combined With Subtrochanteric Transverse Shortening Osteotomy: Factors Associated With Delayed Union at the Osteotomy Site. J Am Acad Orthop Surg Glob Res Rev. 2020 Aug;4(8):e20.00056.

35. Caylak R, Ors C, Togrul E. Minimum 10-Year Results of Cementless Ceramic-On-Ceramic Total Hip Arthroplasty Performed With Transverse Subtrochanteric Osteotomy in Crowe Type IV Hips. J Arthroplasty. 2021 Oct;36(10):3519–26.

36. Erdem Y, Bek D, Atbasi Z, Neyisci C, Yildiz C, Basbozkurt M. Total hip arthroplasty with rectangular stems and subtrochanteric transverse shortening osteotomy in Crowe type IV hips: a retrospective study. Arthroplast Today. 2019 Jun;5(2):234–42.

37. Dallari D, Pignatti G, Stagni C, Giavaresi G, Del Piccolo N, Rani N, Veronesi F, Fini M. Total hip arthroplasty with shortening osteotomy in congenital major hip dislocation sequelae. Orthopedics. 2011 Aug 8;34(8):e328-333.

38. Zhong C, Cai XZ, Yan SG, He RX. S-ROM modular arthroplasty combined with transverse subtrochanteric shortening for Crowe type IV congenital dislocation of hip. Chin Med J (Engl). 2011 Dec;124(23):3891–5.

39. Oe K, Iida H, Nakamura T, Okamoto N, Wada T. Subtrochanteric shortening osteotomy combined with cemented total hip arthroplasty for Crowe group IV hips. Arch Orthop Trauma Surg. 2013 Dec;133(12):1763–70.

40. Howie CR, Ohly NE, Miller B. Cemented total hip arthroplasty with subtrochanteric osteotomy in dysplastic hips. Clin Orthop Relat Res. 2010 Dec;468(12):3240–7.

41. Akıncı O, Turgut A. Long-Term Results of Total Hip Arthroplasty with Step-Cut Osteotomy in Crowe Type IV Dysplastic Hips. Indian J Orthop. 2022 Apr;56(4):672–9.

42. Ahmed E, Ibrahim EG, Ayman B. Total hip arthroplasty with subtrochanteric osteotomy in neglected dysplastic hip. Int Orthop. 2015 Jan;39(1):27–33.

43. Chareancholvanich K, Becker DA, Gustilo RB. Treatment of congenital dislocated hip by arthroplasty with femoral shortening. Clin Orthop Relat Res. 1999 Mar;(360):127–35.

44. Sukur E, Senel A, Ozdemir U, Akman YE, Azboy İ, Ozturkmen Y. Cementless modular total hip arthroplasty with subtrochanteric transverse shortening osteotomy for high hip dislocations. J Orthop Surg Res. 2022 Mar 4;17(1):139.

45. Mimendia I, Lakhani K, Núñez JH, Barro V, Guerra-Farfán E, Collado D, Hernández A. Total hip arthroplasty associated with transverse subtrochanteric shortening osteotomy and conical stem fixation in Crowe type IV hip dysplasia. Musculoskelet Surg. 2023 Sep;107(3):367–72.

46. Ors C, Caylak R, Togrul E. Total Hip Arthroplasty With the Wagner Cone Femoral Stem in Patients With Crowe IV Developmental Dysplasia of the Hip: A Retrospective Study. J Arthroplasty. 2022 Jan;37(1):103–9.

47. Chen M, Luo Z, Zhu C, Wu K, Zhang X, Shang X. A Reliable Femoral Osteotomy in Total Hip Arthroplasty for Hartofilakidis Type C Developmental Dysplasia of the Hip: Proximal Femoral Reconstruction. J Arthroplasty. 2019 Jun;34(6):1162–7.

48. Ravanbod H, Gharanizadeh K, Mirghaderi P, Hassan A, Abolghasemian M. Subtrochanteric Shortening Osteotomy Provides Superior Function to Trochanter Slide Osteotomy in THA for Patients With Unilateral Crowe Type IV Dysplasia at a Minimum of 3 Years. Clin Orthop Relat Res. 2023 Oct 27;

49. Can A, Sarikaya IA, Yontar NS, Erdogan AO, Gorgun B, Erdogan F. High-Riding Congenital Hip Dislocation: THA With Unilateral vs Bilateral Transverse Femoral Shortening Osteotomy. J Arthroplasty. 2018 May;33(5):1432–6.

50. Inoue D, Kabata T, Kajino Y, Ohmori T, Ueoka K, Tsuchiya H. Comparison of mid-term clinical results between cementless and cemented femoral stems in total hip arthroplasty with femoral shortening osteotomy for Crowe type IV hips. Arch Orthop Trauma Surg. 2021 Jun;141(6):1057–64.

51. Kocabiyik A, Misir A, Kizkapan TB, Yildiz KI, Kaygusuz MA, Alpay Y, Ezici A. Changes in Hip, Knee, and Ankle Coronal Alignments After Total Hip Arthroplasty With Transverse Femoral Shortening Osteotomy for Unilateral Crowe Type IV Developmental Dysplasia of the Hip. J Arthroplasty. 2017 Nov;32(11):3449–56.

52. Karaismailoglu B, Karaismailoglu TN. Comparison of Trochanteric Slide and Subtrochanteric Shortening Osteotomy in the Treatment of Severe Hip Dysplasia: Mid-Term Clinical Outcomes of Cementless Total Hip Arthroplasty. J Arthroplasty. 2020 Sep;35(9):2529–36.
